# Fall and Spring Staging in a Refugia‐Dependent Migratory Snake

**DOI:** 10.1002/ece3.73890

**Published:** 2026-07-10

**Authors:** Andrew C. Jesper, Michael J. Dreslik, Scott A. Eckert

**Affiliations:** ^1^ National Great Rivers Research and Education Center East Alton Illinois USA; ^2^ Illinois Natural History Survey Prairie Research Institute Champaign Illinois USA; ^3^ Department of Ecology and Environmental Management Principia College Elsah Illinois USA

**Keywords:** ectotherms, hibernacula, migration, phenology, refugia, staging

## Abstract

Animals reliant on seasonal refugia face timing challenges because refugia can act as temporal bottlenecks that limit access to vital resources including food, mates, and suitable thermal conditions. Consequently, the timing of entry into and exit from refugia carries fitness consequences, particularly for temperate migratory reptiles that must balance the threat of low winter temperatures against short active seasons. How such species coordinate migration with refugia use during thermally unpredictable fall and spring transitions remains poorly understood. One potential strategy is the use of staging periods, temporary pauses near refugia that decouple migration timing from entry into or exit from refugia, allowing individuals to synchronize seasonal transitions with favorable conditions. Although staging is well documented in other migratory taxa, it remains poorly studied in reptiles. Here, we used Timber Rattlesnakes ( *Crotalus horridus* ) to investigate the existence, timing, and function of fall and spring staging in a refugia‐dependent migratory reptile. During the 2021–2022 and 2022–2023 overwintering periods, we observed prolonged fall (average in 2021 = 29 days, 2022 = 8 days) and spring (average in 2022 = 22, 2023 = 26 days) staging, during which snakes remained close to refugia, exhibited limited movement, and entered and exited refugia primarily at night. Movements were consistently associated with two thermal thresholds: on average, ingress into and egress from refugia occurred near ~14°C, whereas fall arrival and spring departure occurred near ~19°C, suggesting temperature may act as an important proximate cue. Consequently, snakes spent over 6 months within refugia, extended to nearly 8 months when staging periods were included, leaving only ~4 months for active‐season activities. Our results provide evidence that prolonged staging represents distinct but phenologically flexible phases, functioning as adaptive buffers that likely help individuals navigate thermally variable seasonal transitions. Recognizing staging as a functional component of refugia use has important implications for understanding phenological risk and climate sensitivity in migratory, refugia‐dependent ectotherms.

## Graphical abstract

Animals reliant on seasonal refugia face strong fitness constraints imposed by the timing of migration relative to thermally unpredictable seasonal transitions. Using Timber Rattlesnakes ( *Crotalus horridus* ), we show that prolonged fall and spring staging periods characterized by restricted movements near refugia and distinct thermal thresholds function as a thermally mediated buffer that decouples migration from refuge entry and exit. Staging represents a previously underappreciated but ecologically important phase of refugia use with implications for phenological risk and climate sensitivity in migratory ectotherms.

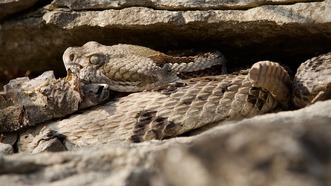

## 1 Introduction

Animals relying on seasonal refugia to survive environmental stress, such as extreme temperatures, drought, or resource scarcity, face a fundamental ecological challenge of timing (Constant et al. 2024; Gregory 1982; Navas and Carvalho 2010). Although seasonal refugia provide critical shelter, they act as temporal bottlenecks by limiting access to essential resources such as food and mates (Geiser 2013; Ultsch 2006). Consequently, the timing of entry into (ingress) and exit from (egress) refugia carries significant fitness consequences. Mistimed use can expose individuals to lethal environmental conditions, increase predation risk, or truncate opportunities for growth, maintenance, and reproduction (Bieber et al. 2018; López‐Alfaro et al. 2013; Willis 2017). The challenge of timing ingress and egress is especially pronounced for temperate reptiles, which undergo prolonged winter dormancy in subterranean refugia to survive freezing temperatures (Gregory 1982; Holden et al. 2021; Turner and Maclean 2022). At high latitudes or elevations, where winter may persist for more than half the year, such individuals must balance the risk of cold exposure with the need to exploit brief active seasons (Blouin‐Demers et al. 2000; DeGregorio et al. 2017). Success depends on anticipating future conditions and fine‐tuning overwintering phenology accordingly (Jesper et al. 2023; DeGregorio et al. 2017).

Timing the use of overwintering refugia becomes more complex when differences in movement ecology are considered, particularly between migratory and nonmigratory strategies (Brown et al. 2023; Southwood and Avens 2010; Seigel et al. 1987). Nonmigratory reptiles generally stay closer to refugia year‐round, enabling rapid ingress or egress in response to proximate cues such as temperature, humidity, and photoperiod (Gregory 1982). Conversely, migratory terrestrial reptiles can travel long distances, typically between 1 and 10 km, between overwintering refugia and disjunct summer habitats (Eckert and Jesper 2024; Harvey and Larsen 2020; Russell et al. 2005). Such species must coordinate refugia use not only with proximate conditions but also with the demands of migration, thereby increasing vulnerability to phenological mismatches (Southwood and Avens 2010). Even minor mistiming, such as arriving at refugia too late in the fall or departing too early in the spring, could result in lethal cold exposure. How migratory and refugia‐dependent reptiles time their movements, particularly during unpredictable fall and spring temperatures, remains poorly understood but is arguably one of the most important decisions in their annual lifecycle.

One potential strategy reptiles might use to coordinate migration timing with safe ingress and egress from overwintering refugia is staging periods. Staging periods, also called transient or stopover periods, are transitional phases where animals temporarily pause seasonal movements to rest, refuel, or synchronize habitat shifts with favorable conditions. Such intermediary phases are widespread across migratory taxa, including birds (Cohen et al. 2021; Schofield et al. 2018; Warnock 2010), fish (Smith 2012), mammals (Fryxell and Sinclair 1988; Lacki et al. 2015), and insects (Chapman et al. 2015; Tenger‐Trolander et al. 2023). For example, birds use staging sites to recover energy and align long flights with favorable temperature and wind regimes (Cochran et al. 2004; Ramenofsky et al. 2008; Schofield et al. 2018). Salmon and other anadromous fish accumulate energy at key locations before continuing upstream migrations (Smith 2012), whereas ungulates and bats often aggregate at temporary sites to optimize foraging or thermoregulation (Custer et al. 2023; Fryxell and Sinclair 1988; Lacki et al. 2015). In short, staging periods help animals balance trade‐offs between movement, resource acquisition, and survival under variable environmental conditions. Although rarely documented in reptiles, analogous periods could provide similar benefits by buffering against environmental unpredictability: using overwintering and stopover habitats to fine‐tune migration timing and enhance fitness in seasonal landscapes.

Limited evidence, primarily from temperate snakes, suggests that staging periods can occur after fall migration prior to refugia ingress, and after spring egress before departure to summer habitats. Several species, including Timber Rattlesnakes ( *Crotalus horridus* ), Common European Vipers ( *Vipera berus* ), and Eastern Montpellier snakes ( *Malpolon insignitus* ), reportedly remain near refugia for weeks or months, suggesting distinct phenological phases with potential adaptive significance (e.g., Brown 1992; Dyugmedzhiev et al. 2019; Martin 1992; Viitanen 1967). While staging periods may facilitate ecdysis (Brown 1993), mating (primarily in spring; Shine et al. 2001), foraging/digestion (Martin 1992; Sealy 2002), or physiological regulation (Angilletta Jr. 2009; Gregory 1982), the most compelling hypothesis is they serve as temporal buffers, allowing individuals to align refugia use and migration with favorable environmental conditions, particularly temperature (Brown 1992; Martin 1992). Specifically, in the fall, individuals may arrive early at refugia, anticipating cold conditions, but delay ingress to extend opportunities for active season activities. In spring, they may emerge during warm periods while remaining near refugia, delaying departure until temperatures consistently exceed thermal minima (Martin 1992). Such behaviors suggest staging periods may be an adaptive response to climatic uncertainty, though the evidence is mainly anecdotal, and questions about their duration, function, and even existence remain unresolved.

Here, we used Timber Rattlesnakes ( *C. horridus* ), a pitviper native to the eastern United States, as a model to investigate the existence, phenology, and potential function of fall and spring staging periods in refugia‐dependent and migratory reptiles. *Crotalus horridus* is well suited for such research because individuals make predictable seasonal migrations (1–3 km) between distinct summer and overwintering habitats, exhibit strong fidelity to communal hibernacula, and spend much of the year in brumation, especially in northern and montane populations (Brown 1992; Eckert and Jesper 2024; Martin 2002; Petersen et al. 2019). Studies suggest the species exhibits both fall and spring staging behaviors, but their timing, behavioral context, and ecological role remain unclear (Brown 1992; Martin 1992). Staging habitat typically occurs in ecotones between closed‐canopy woodland and exposed rocky slopes or bluff lines adjacent to refugia, where snakes reportedly balance thermoregulation with concealment (Brown 1993; Jesper et al. 2023, 2024). Reports vary widely on duration, with some noting staging lasts over a month, whereas others report staging is absent altogether (Brown 1992; Martin 1992). Such variation suggests staging may represent a flexible behavioral strategy rather than a fixed annual phase, possibly reflecting interannual or regional thermal conditions.

Our objectives were to: (1) determine whether *C. horridus* exhibit fall and spring staging; (2) describe the phenology, movements, and behaviors associated with each period; and (3) evaluate whether staging periods function as adaptive buffers that may be thermally mediated, which decouple migration timing from refugia entry and exit and allow individuals to navigate unpredictable fall and spring temperatures. Our findings elucidate an overlooked but critical phase in the annual cycle of *C. horridus* and similar species and provide broader insights into adaptations of migratory, refugia‐dependent reptiles to seasonal climatic variability.

## 2 Materials and Methods

### 2.1 Study Site and Data Collection

We examined fall and spring staging periods in a population of *C. horridus* at Principia College in Jersey County, west‐central Illinois, USA. The study site is an upland mesic forest bordered to the south by limestone bluffs along the Mississippi River. Vegetation consists of a matrix of remnant hill prairies and oak‐hickory woodlands. The ~7 km of bluff front encompasses all prominent outcrops on the property, providing crevices, talus, and holes for overwintering refugia. Observations from the 1930s established the presence of refugia, with subsequent studies identifying specific entrances using visual encounter surveys (Jesper et al. 2024), wildlife cameras (Jesper et al. 2023), and VHF radiotelemetry (Eckert and Jesper 2024).

**FIGURE A1:**
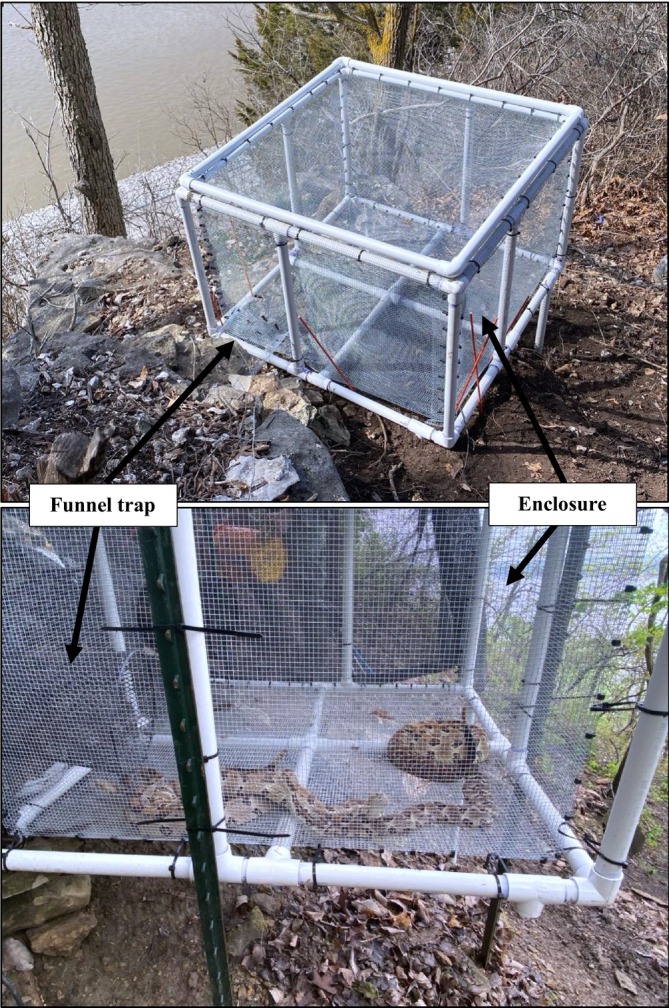
Photographs of a funnel trap and enclosure system used to capture emerging Timber Rattlesnakes ( *Crotalus horridus* ) at refugia in Jersey County, Illinois, during spring egress 2021. A team of researchers visited dens daily to retrieve snakes which had emerged from overwintering refugia, moved through the funnel traps, and became trapped in enclosures.

All fieldwork was conducted under approved Illinois Department of Natural Resources (IDNR) Endangered and Threatened Species permits 15–009, 05‐11S, 4956, 6573, 7304, HSCP 19–04, and 14,636, with all animal handling and surgical procedures approved by the University of Illinois Institutional Animal Care and Use Committee (IACUC) under protocols 22,167 and 22,168. From 2015 to 2021, we captured snakes at refugia using visual encounter surveys during suspected fall ingress (September–October) and spring egress (April–May), as well as opportunistically throughout the active season. In spring 2021, we temporarily installed enclosure traps at refugia entrances (PVC frames ~1.5 m^3^ enclosed with galvanized steel mesh; Figure A1) and checked them daily to supplement initial captures of emerging snakes. We removed the enclosure traps following spring 2021 and did not use them during subsequent monitoring of ingress, egress, or staging behavior, ensuring that movements analyzed in this study were not influenced by trapping infrastructure. All captured snakes were weighed using digital or spring scales, measured for snout–vent length (SVL) and tail length, and sexed using cloacal probing. Each snake also received a Passive Integrated Transponder (PIT) tag (BioMark Inc., Boise, ID) implanted subcutaneously in the distal one‐third of the body.

We selected a subset of captured snakes for VHF radiotelemetry, limiting transmitter mass to ≤ 5% of body mass in accordance with established telemetry guidelines for reptiles (Reinert and Cundall 1982; Weatherhead and Blouin‐Demers 2004), while ensuring approximate representation of both males and females. Veterinarians at the Saint Louis Zoo surgically implanted temperature‐sensitive radio transmitters (Holohil SI‐2 T, Carp, Ontario; battery life 24 months) into the peritoneal cavity of each snake under isoflurane anesthesia (Reinert and Cundall 1982). Snakes were then held for postoperative recovery and released at their capture sites within 1 week of surgery. We acknowledge that surgical implantation procedures could potentially influence behavior during postsurgical recovery. However, we do not believe such effects influenced behavior associated with staging periods examined here because: (1) snakes had a full active season (May–October) to recover in the field prior to the first staging period analyzed (Fall 2021), and (2) all snakes appeared fully healed upon visual inspection of incision sites during subsequent staging periods.

**FIGURE 1:**
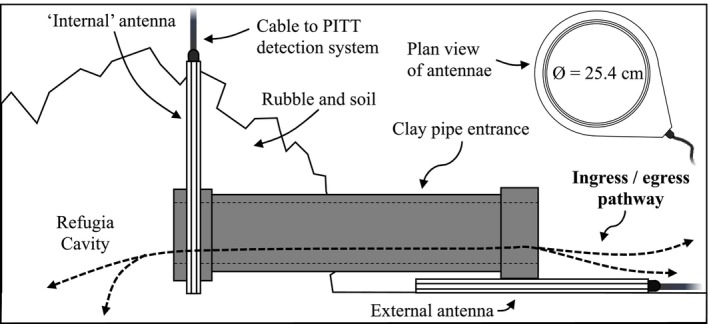
Side‐view diagram (not to scale) of a modified Timber Ratttlesnake ( *Crotalus horridus* ) refugium entrance fitted with a small‐scale PIT tag detection system. Each PIT detection system consisted of a BioMark IS1001‐12 V PIT tag reader and circular antennae (25.4 cm internal diameter; 15 m cable length). Three PIT tag systems operated at three refugia in Jersey County, Illinois, with two antennae positioned at known entrances and integrated with clay pipe and surrounding rubble/soil to minimize bypass around the antennae and improve detection consistency during ingress and egress movements.

Two complementary approaches characterized fall and spring staging periods. First, we monitored PIT‐tagged individuals at three communal refugia using PIT tag detection systems (Biomark Inc., Boise, ID; hereafter “PITT systems”). Each PITT system included an IS1001‐12 V reader connected to circular antennae (25.4 cm internal diameter; 15 m cable length). At each monitored refugium, we installed PIT tag antennae at primary entrances used by snakes (Figure 1), identified through repeated visual encounter surveys, radiotelemetry observations, and trail camera photos documenting recurring ingress and egress activity from previous studies (e.g., Eckert and Jesper 2024; Jesper et al. 2023). We inserted a clay pipe (15 cm × 60 cm) fitted with antennae at both ends and partially buried the pipe and surrounding antennae in soil and limestone rubble to simulate natural conditions while minimizing bypass around monitored entrances. Dual antenna placement allowed us to determine movement direction (ingress or egress). Although additional undocumented entrances may have existed within the broader talus system, repeated telemetry observations and PIT detections indicated snakes consistently used the monitored entrances during ingress and egress. PITT systems operated continuously throughout the overwintering period.

Second, we tracked all snakes equipped with VHF radio transmitters during spring egress and fall ingress. During both periods, we located snakes daily using a three‐element folding yagi antenna and receiver, with locations recorded using a Garmin GPSMAP 66i GPS or an Apple iPad paired with Bluetooth GPS. We visually confirmed snake locations during each tracking event, and reused the same coordinates when snakes remained in the exact same location across multiple days to reduce GPS error that could falsely suggest movement.

**FIGURE 2:**
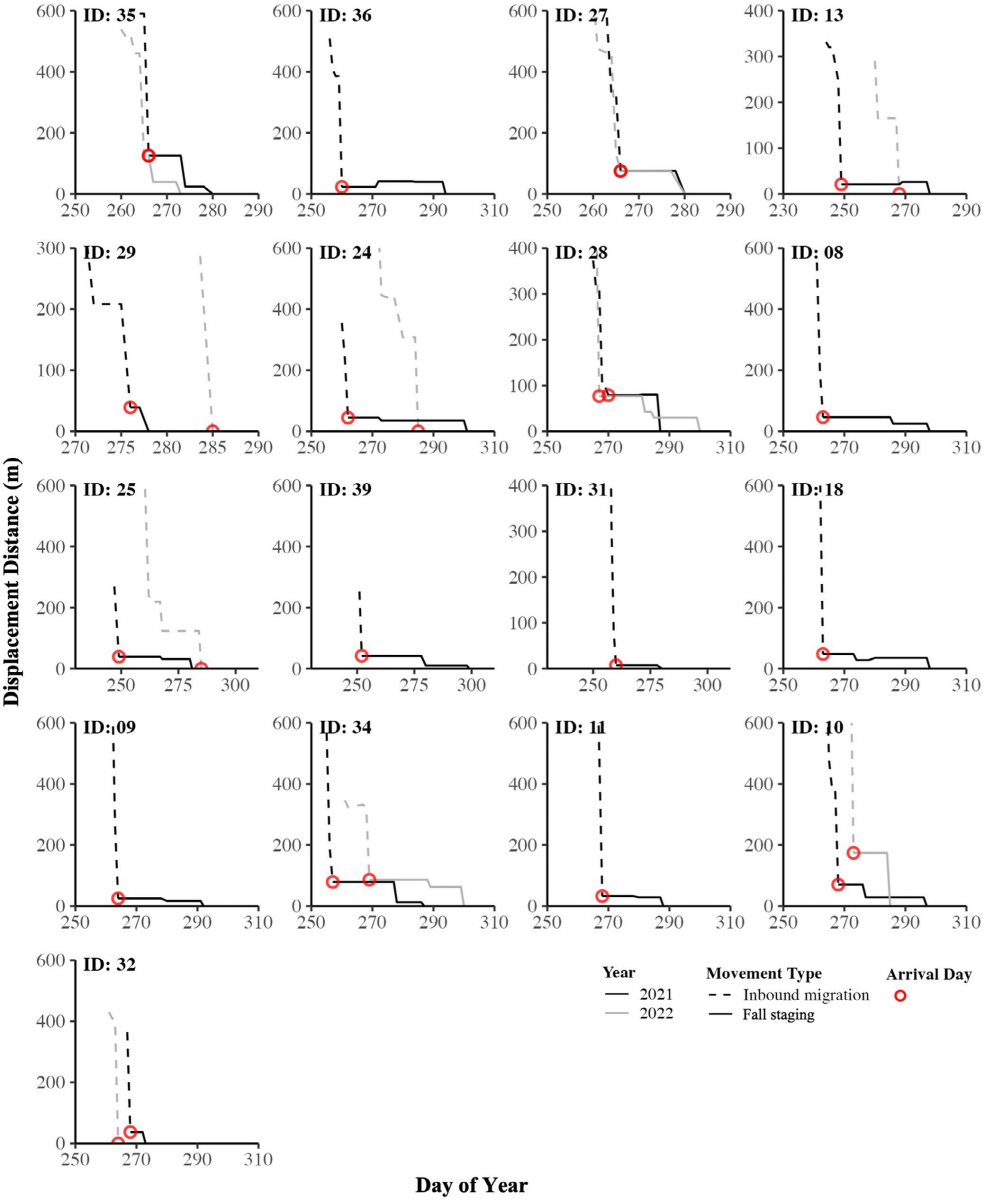
Maximum Euclidean distance (“displacement”) from overwintering refugia by 17 Timber Rattlesnakes ( *Crotalus horridus* ) during the fall of 2021 and 2022 prior to entry into refugia (27 snake‐year combinations).

**FIGURE 3:**
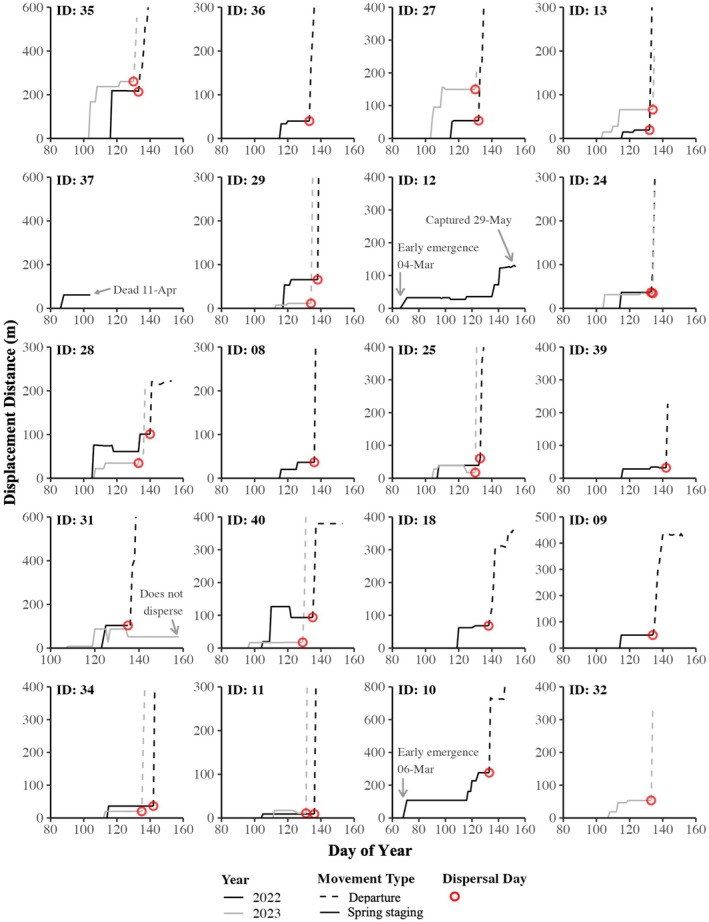
Maximum Euclidean distance (“displacement”) from overwintering refugia by 20 post‐emergent Timber Rattlesnakes ( *Crotalus horridus* ) during the spring of 2022 and 2023 (31 snake‐year combinations).

We used straight‐line Euclidean distances (“displacement”) between daily snake locations and refugia to determine transitions between long‐distance directional migration and localized staging behavior associated with overwintering habitat (Figures 2 and 3). In spring, we began tracking snakes after they emerged from refugia (identified via the PITT system or daily VHF telemetry) and continued until they departed for summer habitat. We defined departure using the displacement trajectories as the first in a series of long‐distance, continuous, directional movements away from bluff habitat into the interior old‐growth forest, marking an abrupt transition from localized residency near refugia to outbound migration (see red circles in Figure 3).

In fall, we initiated daily checks of snakes once individuals came within telemetry range of observers stationed near the refugia (typically ~600 m away; Figure 2). We then tracked snakes daily as they continued inbound migratory movements toward the distinct south‐facing bluff habitat that differed markedly from the surrounding upland forest. Mirroring the spring departure transition but in reverse, we defined arrival at overwintering habitat using displacement trajectories as the day on which long‐distance directional migratory movements terminated within the south‐facing bluff habitat, marking a clear transition from inbound migration to localized residency near refugia (see red circles in Figure 2). We then continued tracking until ingress into refugia was confirmed via the PITT systems or VHF telemetry.

### 2.2 Analysis

We defined the fall staging period as the number of days between a snake's arrival at overwintering refugia and its ingress into a refugium, and the spring staging period as the number of days between egress and subsequent departure toward summer habitat. We compiled dates for each movement type (fall: arrival and ingress; spring: egress and departure) for all monitored snakes, determined from VHF telemetry and PITT data.

We used two linear mixed models (LMMs) to quantify the timing and duration of fall and spring staging periods in R using the nlme package (Pinheiro and Bates 2023) one for fall (*fall_day* model) and one for spring (spring_day model). For each model, we treated ordinal date (day of year) as the response variable, with movement type and year as interacting fixed effects. Models were fit assuming Gaussian error distributions with an identity link. We included snake ID as a random intercept and slope to account for repeated measures across years and a partially paired dataset, because tracking methods varied among individuals. The mixed‐effects structure also accommodated unbalanced repeated‐measures data, allowing all available observations to be included without requiring paired fall arrival and ingress dates or paired spring egress and departure dates for each snake within a given season (Zuur et al. 2009). Although unpaired observations did not contribute information on within‐individual staging intervals, they still informed overall ingress timing and interannual phenological variation and were therefore retained in the mixed‐effects models. Significant movement type × year interactions indicated phenological flexibility across years, while nonsignificant interactions suggested more fixed schedules. We evaluated coefficient informativeness using 95% confidence intervals (CIs), estimated marginal means (±95% CI) using the emmeans package (Lenth 2023), and tested for differences between estimated marginal means using pairwise contrasts. We then visualized the results using the ggplot2 package (Wickham 2016).

To characterize behavior during each staging period, we summarized VHF telemetry data as the maximum Euclidean distance from refugia, the mean number of locations used, and the mean duration of stay per location. We calculated the metrics using ArcGIS Desktop 10 (ESRI 2011) and analyzed them with LMMs in lme4 (Bates et al. 2015), with snake ID as a random effect. All response variables met assumptions of normality and homogeneity of variance without requiring transformation. We report model intercepts (±95% CI) representing population means and did not test for sex or size effects due to the limited sample sizes.

To assess whether temperature influenced movement timing, we analyzed the minimum daily temperatures (hereafter “temperature” unless otherwise stated) at which snakes performed each movement type (fall: arrival and ingress; spring: egress and departure). Two additional LMMs (*fall_temp* and *spring_temp*) followed the same model structure and approach as above, including snake ID as both a random intercept and slope. The models treated minimum daily temperature as the response variable and included year and movement type as interacting explanatory variables. Minimum daily temperature likely best represents the lower thermal conditions experienced by snakes during staging periods and may therefore be more biologically relevant to movement timing than mean or maximum temperatures (e.g., Brown 1992; Angilletta Jr. 2009). A weather station located ~1 km from the refugia on the Principia College campus (Station ID: KILELSAH3) provided the daily minimum temperatures we used for the analysis. Informative movement type × year interactions indicated movements occurred at different temperatures across years, suggesting nonthermal drivers, whereas non‐informative interactions indicate movements occurred at consistent temperatures between years, indicating thermally regulated timing. We again used estimated marginal means and pairwise contrasts between them for interpretation.

Residual diagnostics indicated whether model assumptions were satisfied in all models. Residual normality was assessed using Q–Q plots and Shapiro–Wilk tests, and homogeneity of variance was assessed using residual‐versus‐fitted plots, Pearson residual plots, and Levene's tests (Pinheiro and Bates 2000; Zuur et al. 2009). When heteroskedasticity associated with movement type or year was detected, models were refit using a within‐group variance structure implemented with the varIdent function in the nlme package, which allows residual variances to differ among factor levels (Pinheiro and Bates 2000; Zuur et al. 2009). Use of group‐specific variance structures is recommended for mixed‐effects models when residual variance is nonconstant, as failure to account for heteroskedasticity can bias parameter estimates and statistical inference (Bolker et al. 2009). We reassessed model adequacy following refitting and based all subsequent inference on variance‐corrected models; no response variables required transformation to meet assumptions.

## 3 Results

### 3.1 Overview

We monitored *C. horridus* using PITT systems and VHF radiotelemetry for one or more seasons from fall 2021 through spring 2023, encompassing two fall and two spring seasons. We deployed PITT systems across three communal refugia, two of which were 0.6 km apart and a third approximately 4 km to the west. Through repeated monitoring since 2015, we identified a single primary entrance at two refugia and three primary entrances at a third refugium (~3 m apart). We equipped all primary entrances with PITT systems that continuously monitored snake ingress and egress from approximately 1 September through 1 June of the following year.

Across the study period, we monitored 38 snakes (17 females, 21 males), which had a mean body mass of 792 g (SD = 472) and mean SVL of 93.7 cm (SD = 23.8). Of these, 20 individuals (12 males, 8 females) were equipped with VHF transmitters. Mean body mass and SVL of VHF‐tracked snakes were 865 g (SD = 417) and 95.0 cm (SD = 25.3), respectively. All VHF‐tracked snakes used refugia monitored by the PITT systems and were therefore monitored using both VHF telemetry and PIT‐tag detection, whereas the remaining 18 snakes were monitored exclusively via PIT‐tag detections. We obtained 2488 telemetry locations during the two staging periods, including 732 fall locations (mean = 43.1 locations per snake per season, SD = 17.6, range = 11–80) and 1756 spring locations (mean = 87.8 locations per snake per season, SD = 24.5, range = 27–114). Because telemetry was conducted daily throughout transient periods, telemetry effort varied among individuals according to the duration of staging behavior exhibited by each snake. All reported results are based on variance‐corrected models following residual diagnostics.

### 3.2 Fall Staging Period

After excluding three snakes exhibiting aberrant overwinter behavior (see below), the fall dataset included 27 paired seasonal records from 17 VHF‐tracked snakes in which both arrival to overwintering habitat and subsequent ingress into refugia were documented within the same year (17 records in 2021; 10 records in 2022). Additional unpaired observations from PIT‐tag monitored snakes in which only ingress was documented increased the dataset to 46 total ingress events from 28 snakes.

**FIGURE 4:**
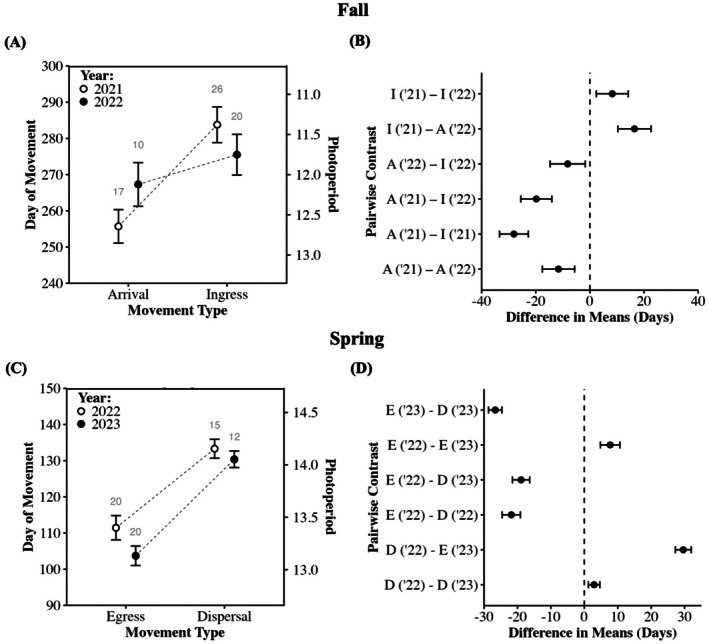
Marginal (mean) day of fall and spring staging‐related movements by Timber Rattlesnakes ( *Crotalus horridus* ) in Jersey County, Illinois. Fall plots show the marginal day of arrival to overwintering habitat (“Arrival”) and ingress into refugia (“Ingress”) for 2021 and 2022 (subplot A), alongside pairwise contrasts comparing movement timing between years and movement types (subplot B; y‐axis labels: A, Arrival; I, Ingress). Spring plots show the marginal day of egress from refugia (“Egress”) and departure to summer habitats (“Departure”) for 2022 and 2023 (subplot C), alongside pairwise contrasts comparing movement timing between years and movement types (subplot D; y‐axis labels: E, Egress; D, Departure). Error bars represent 95% confidence intervals. subplot A and C include sample sizes for each group and a secondary axis showing corresponding photoperiod values for reference.

Snakes consistently arrived at overwintering habitat well before ingress into refugia, resulting in prolonged fall staging periods that differed between years (Table 1). In 2021, snakes arrived an estimated 29.23 days before ingress (Figure 4; marginal mean arrival = day 256, 13 September, 95% CI = day 251–260; marginal mean ingress = day 285, 12 October, 95% CI = day 279–289), whereas in 2022 the staging interval was shorter, averaging 8.2 days (marginal mean arrival = day 267, 24 September, 95% CI = day 261–273; marginal mean ingress = day 276, 3 October, 95% CI = day 270–281). In addition to these within‐year differences, both arrival and ingress dates differed between years (Figure 4).

**TABLE 1:** Parameter estimates for four linear mixed models (LMMs) used to examine the timing (day of year) and thermal conditions (minimum daily temperature) associated with seasonal staging movements of 38 Timber Rattlesnakes ( *Crotalus horridus* ) in Jersey County, Illinois. Models evaluated: (1) Day of arrival and ingress into refugia in fall (“fall_day”), (2) day of egress and departure to summer ranges in spring (“*spring_day*”), (3) minimum daily temperature during fall arrival and ingress (“*fall_temp*”), and (4) minimum daily temperature during spring egress and departure (“*spring_temp*”). Day of year or minimum daily temperature was included as the response variable, with movement type (Fall = arrival and ingress; Spring = egress and departure) and study year included as explanatory variables with interaction terms. Columns represent the model parameter (Parameter), parameter estimate (Estimate), and upper and lower 95% confidence intervals (CI).

Parameter	Estimate	Lower 95% CI	Upper 95% CI
*fall_day model*			
Intercept	255.71	259.34	252.07
Movement type	28.06	33.38	22.74
Year	11.59	17.56	5.62
Movement type × year	−19.86	−11.47	−28.25
*Spring_day model*			
Intercept	110.58	113.51	107.65
Movement type	21.88	24.59	19.17
Year	−7.76	−4.8	−10.73
Movement type × year	4.44	7.73	1.14
*fall_temp* model			
Intercept	19.7	20.93	18.47
Movement type	−5.18	−3.04	−7.33
Year	−0.52	1.5	−2.55
Movement type × year	−0.49	2.86	−3.83
*Spring_temp* model			
Intercept	14	15.93	12.06
Movement type	5.63	7.75	3.51
Year	−0.09	1.98	−2.16
Movement type × year	−1.22	1.33	−3.76

**FIGURE 5:**
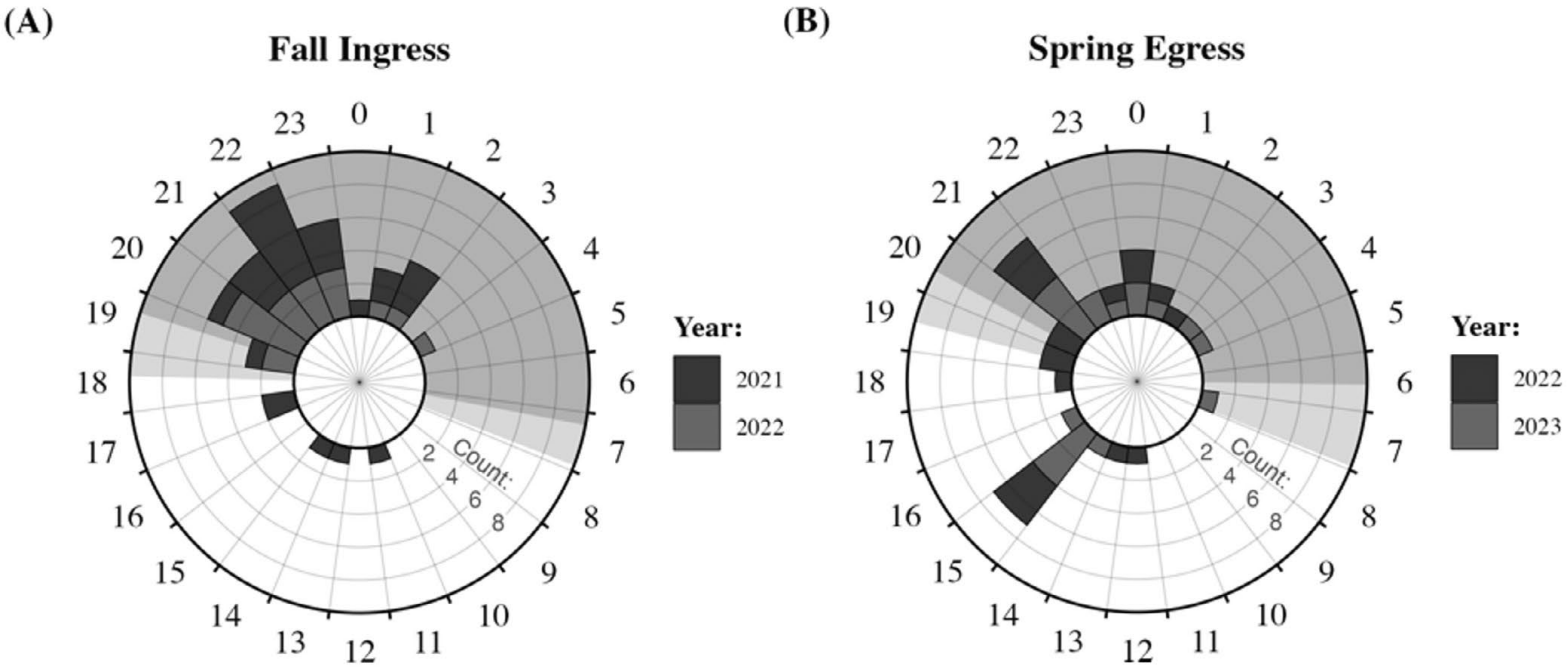
The time of fall ingress into refugia (subplot A; *n* = 46 ingress events) and spring egress from refugia (subplot B; *n* = 40 egress events) by PIT tagged Timber Rattlesnakes ( *Crotalus horridus* ) in Jersey County, Illinois. Each plot represents a 24‐h circular “clock” (e.g., 0 = 12 a.m.) with gray bars showing the number of ingress/egress events observed within each hour. Data pooled across refugia. Shaded sections depict hours of darkness (dark gray) and dawn/dusk (mid gray)—data collected using BioMark Passive Integrated Transponder (PIT) tag detection systems.

**FIGURE A2:**
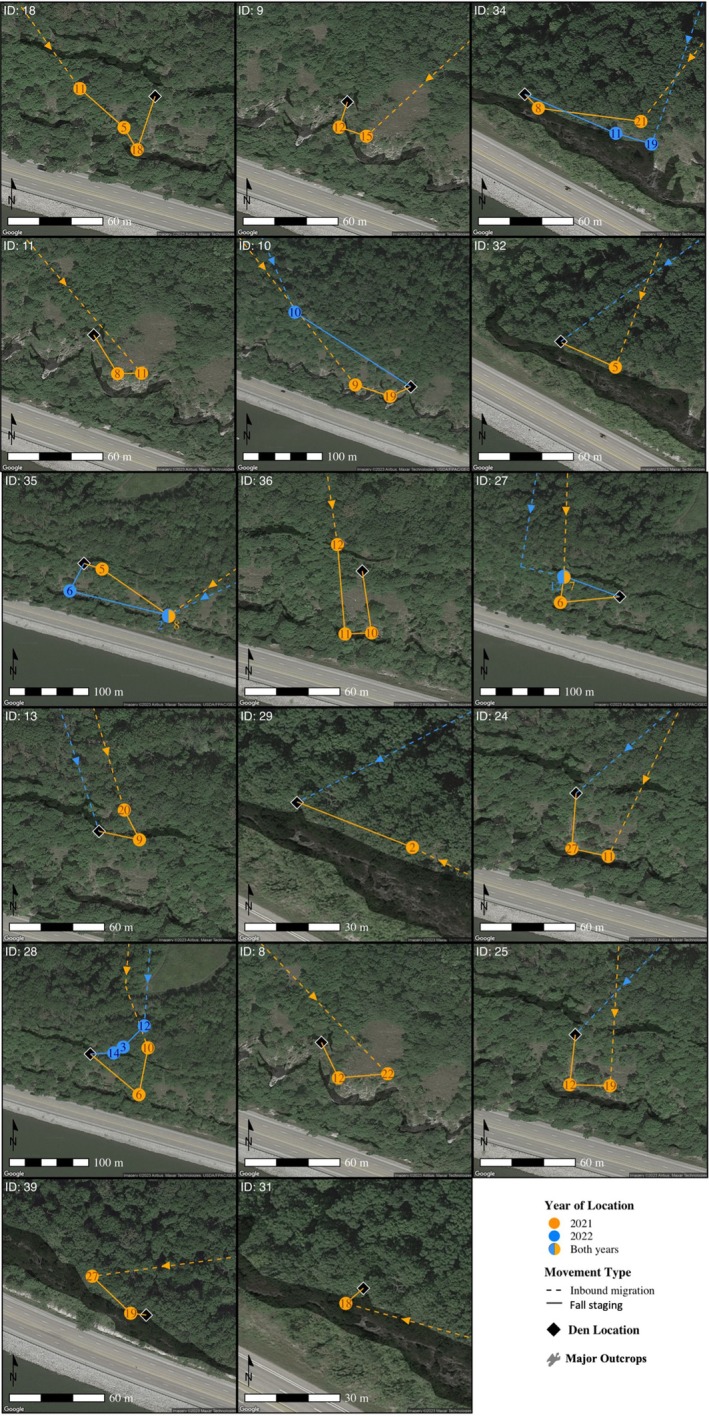
Staging movements of 17 Timber Rattlesnakes ( *Crotalus horridus* ) between arrival from summer habitats and ingress into overwintering refugia during the fall of 2021 and 2022 (27 snake‐year combinations). Numbers within each circle represent the duration (in days) snakes spent at a given location. Lines connect sequential movements of each snake with arrows indicating the direction of movement. Prominent outcrops (translucent black polygons) are shown as reference.

**FIGURE A3:**
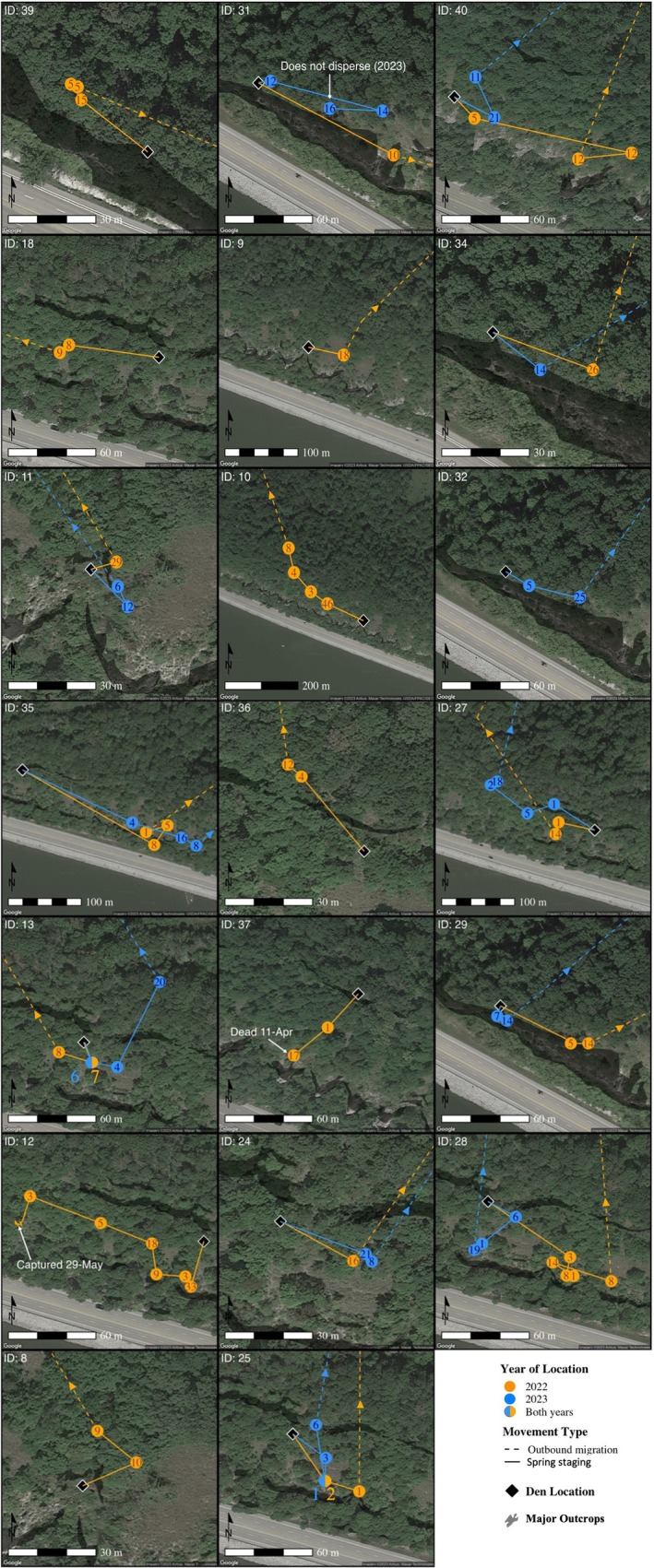
Post‐egress “staging” movements from 20 Timber Rattlesnakes ( *Crotalus horridus* ) from emergence from refugia to departure to summer habitats in the spring of 2022 and 2023 (31 snake‐year combinations). Individual snakes are color‐coded with numbers representing the duration (in days) each snake spent at a given location. Lines connect sequential movements of each snake with arrows indicating the direction of the first “long‐distance” departure/outbound migratory movement. Prominent outcrops (black polygons) are shown as reference.

Of the 27 paired tracking records from 17 snakes, six ingressed into refugia on the same day they arrived (hence did not stage prior to entering hibernation), whereas 21 remained on the surface before ingress. Surface‐active snakes concealed themselves under leaves, rocks, or hollow logs close to refugia (mean distance from refugia = 58.17 m; CI = 39.67–76.23 m, Figures A2 and A3), moved little during the staging period, occupying an average of 1.91 locations (CI = 1.65–2.17, range = 1–8) and remained at each location for an average of 12.29 days (CI = 4.75–19.88, range = 1–26). Snakes entered refugia primarily at night (Figure 5), with a noticeable increase in activity beginning after sunset. Of the 28 snakes detected via PITT systems, 19 entered refugia each fall and did not reemerge until the following spring. Nine snakes reemerged shortly after ingress during one or both years before reentering refugia later the same day and remaining underground until spring. Three snakes shuttled frequently between refugia and the surface over multiple days, accounting for 59 shuttling events across one or both overwintering periods. The three snakes, one with a visible injury around the incision site of the VHF transmitter (snake ID 10) and two which were later found dead (snake IDs 12 and 37), typically emerged in the afternoon (mean time = 01:30 p.m.; SD = 1.87 h.) and reentered refugia in the evening of the same day (mean time = 05:18 p.m.; SD = 2.21 h.). We therefore censored snakes 10, 12, and 37 from further analysis because physiological impairment and aberrant behavior confounded movement patterns.

**FIGURE 6:**
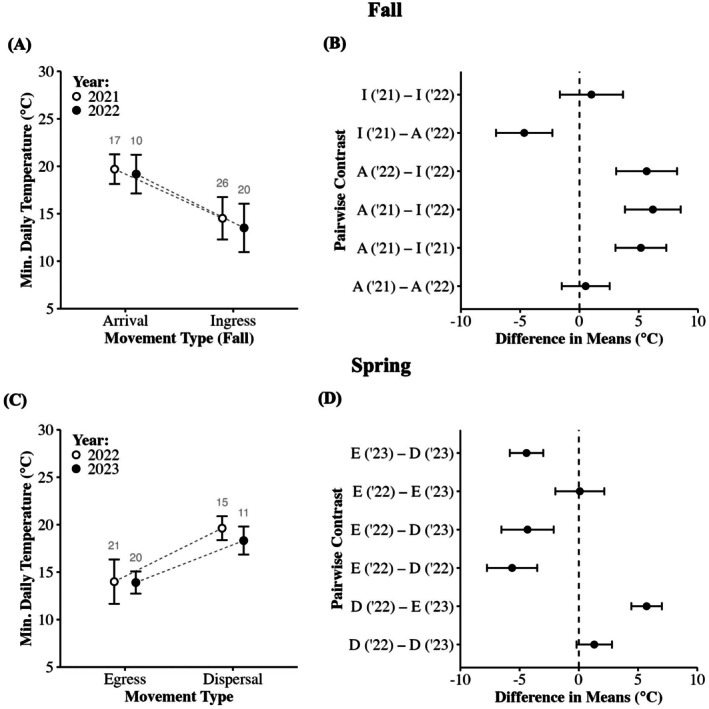
Marginal (mean) minimum daily temperature associated with fall and spring staging‐related movements by Timber Rattlesnakes ( *Crotalus horridus* ) in Jersey County, Illinois. Fall plots show the marginal minimum daily temperature associated with arrival to overwintering habitat (“Arrival”) and ingress into refugia (“Ingress”) for 2021 and 2022 (subplot A), alongside pairwise contrasts comparing fall movement timing between years and movement types (subplot B; y‐axis labels: A, Arrival, I, Ingress). Spring plots show the marginal minimum daily temperature associated with egress from refugia (“Egress”) and departure to summer habitats (“Departure”) for 2022 and 2023 (subplot C), alongside pairwise contrasts comparing spring movement timing between years and movement types (subplot D; y‐axis labels: E, Egress, D, Departure). Error bars represent 95% confidence intervals. Panels A and C include sample sizes for each group.

**FIGURE 7:**
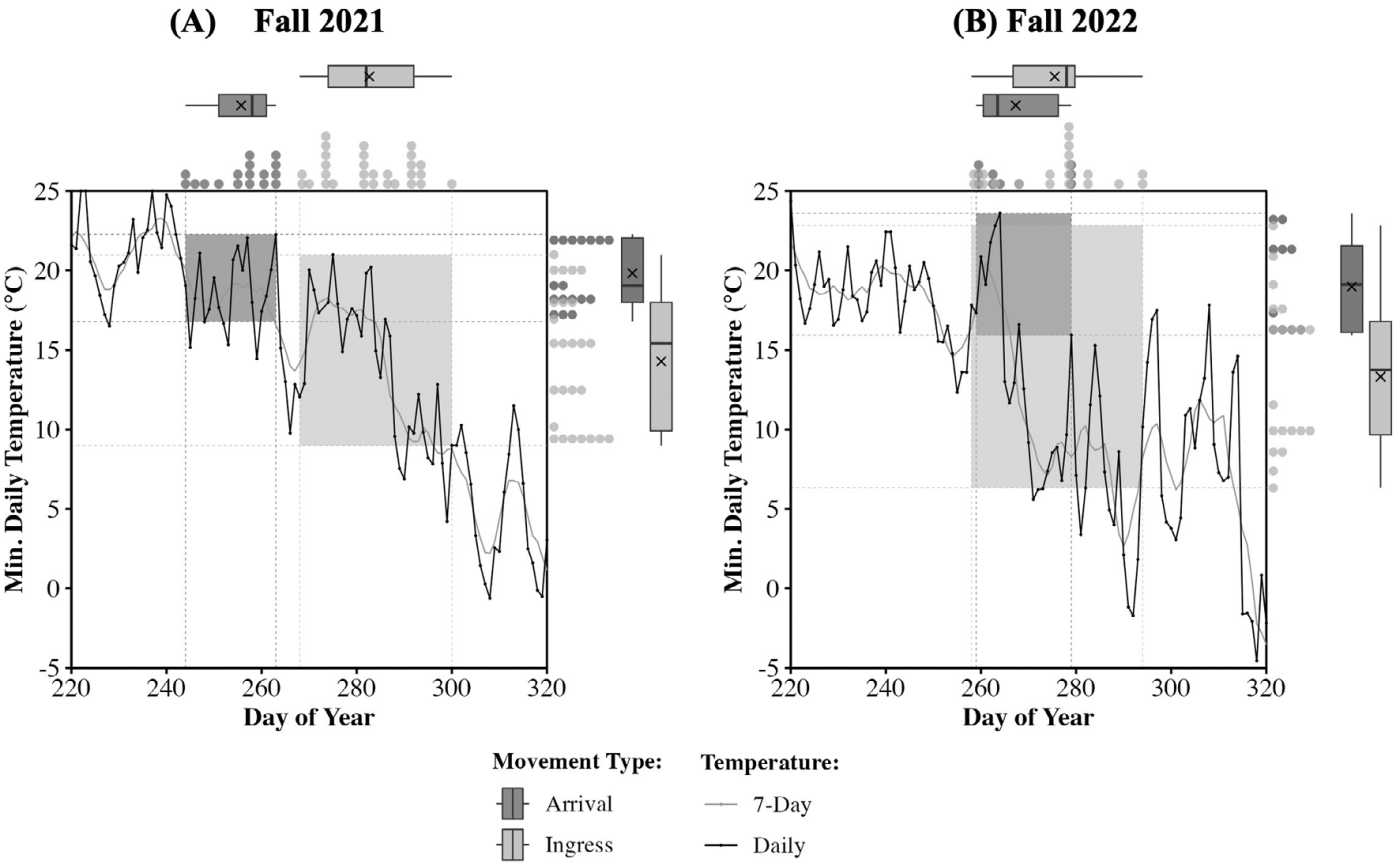
The daily minimum temperature and day of arrival to overwintering habitat and ingress into refugia by Timber Rattlesnakes ( *Crotalus horridus* ) in Jersey County, Illinois, during fall 2021 (subplot A) and 2022 (subplot B). Dot and box plots display the distributions of arrival and ingress day and minimum daily temperature for all monitored snakes (top and right, respectively). Each boxplot displays the range (whiskers), interquartile range (boxes; middle 50% of data), median (vertical line), and mean (cross) of the data for each group. The bin size for each dot plot was set to 1‐unit increments (i.e., 1 day or 1°C). Translucent boxes (and dashed lines) spanning the data range for both axes are shown for reference.

Snakes arrived at overwintering habitat at substantially warmer temperatures than those at which they ultimately entered refugia (Figure 6; Table 1). Across both years, arrival occurred near 19.0°C (2021 mean = 19.7°C, 95% CI = 18.1°C–21.3°C; 2022 mean = 19.2°C, 95% CI = 17.1°C–21.2°C), whereas ingress occurred on average near 14.0°C (2021 mean = 14.2°C, 95% CI = 12.3°C–16.8°C; 2022 mean = 13.5°C, 95% CI = 11.0°C–16.1°C), representing a difference of approximately 5°C. Arrival and ingress temperatures were similar between years. Visual examination of the fall temperature profiles of both years indicated snakes began entering refugia only after a rapid decline in temperature (Figure 7). In fall 2021, all monitored snakes had arrived at overwintering sites before the temperature decreased (~Day 265; Figure 7A); thus, there was no overlap between arrival and ingress days. Conversely, in 2022, the temperature sharply decreased as snakes were still arriving, so the arrival and ingress dates overlapped considerably (Figure 7B).

### 3.3 Spring Staging Period

After excluding three snakes exhibiting the aberrant overwinter behavior, the spring dataset included 31 paired seasonal records from 20 VHF‐tracked snakes in which both egress from refugia and subsequent departure to summer habitats were documented within the same year (18 records in 2022; 13 records in 2023). Additional PIT‐monitored snakes for which only ingress was documented increased the dataset to 46 total ingress events from 28 snakes. Reduced sample sizes relative to fall primarily resulted from mortality or VHF transmitter failure.

Snakes consistently remained near overwintering refugia for weeks after emergence before departing to summer habitats, resulting in prolonged spring staging periods (Table 1; Figure 4). In 2022, snakes departed an estimated 21.81 days after egress (marginal mean egress = day 111, 21 April, 95% CI = day 107–114; marginal mean departure = day 133, 13 May, 95% CI = day 130–135). In 2023, the staging interval was longer, averaging 26.35 days (marginal mean egress = day 103, 13 April, 95% CI = day 100–106; marginal mean departure = day 129, 9 May, 95% CI = day 127–132). In addition to these within‐year differences, both egress and departure dates shifted between years, although the difference in departure timing was relatively small (3 days).

**FIGURE 8:**
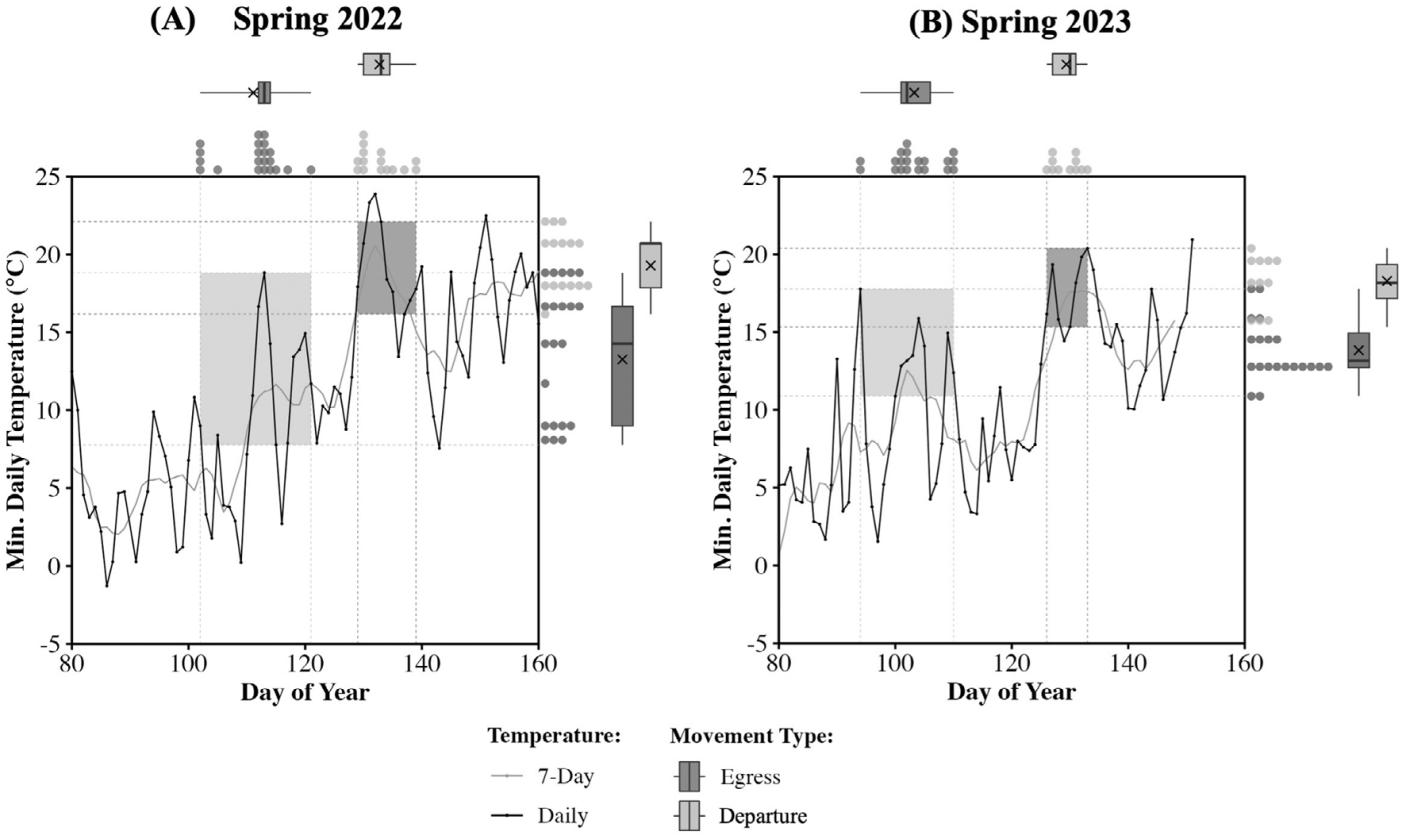
The minimum daily temperature and day of egress from refugia and subsequent departure to summer habitats by Timber Rattlesnakes ( *Crotalus horridus* ) in Jersey County, Illinois, during spring 2022 (subplot A) and 2023 (subplot B). Dot and box plots display the distribution of data for the day (top) and minimum daily temperature (right) for egress and departure by all monitored snakes. Each boxplot displays the range (whiskers), interquartile range (boxes; middle 50% of data), median (vertical line), and mean (cross) of the data for each group. The bin size for each dot plot is set to 1‐unit increments (i.e., 1 day or 1°C). Translucent boxes (and dashed lines) spanning the data range for both axes are shown for reference.

Egress in both years occurred under highly stochastic spring temperatures, producing a staggered emergence pattern in which groups of snakes emerged during warm periods before colder conditions temporarily precluded activity (Figure 8). PIT‐tag detections showed that snakes emerged primarily at night, with a smaller midday peak (Figure 5B), and did not return to refugia even during highly variable post‐emergent temperatures. Upon emergence, all VHF‐equipped snakes moved to nearby south‐facing bluff habitat (Figure 3; Figure A3), where they concealed themselves beneath leaves, rocks, and hollow logs near overwintering refugia until departure (mean distance = 75.50 m; CI = 45.3–105.9). Like fall staging, snakes exhibited limited movement, occupying an average of 2.4 locations (CI = 1.96–2.93; range = 1–6) and remaining at each location for an average of 10.63 days (CI = 8.74–12.52; range = 1–46). Several snakes returned to the same staging locations used during the preceding fall or in previous years (Figure A3).

Like fall ingress, spring movements followed distinct thermal thresholds, with snakes emerging from refugia at cooler temperatures than those at which they departed for summer habitat (Figure 6; Table 1). Across both years, egress occurred near 14.0°C (2022 mean = 14.1°C, 95% CI = 11.6°C–16.3°C; 2023 mean = 14.0°C, 95% CI = 12.75°C–15.1°C), whereas departure occurred near 19.0°C (2022 mean = 19.7°C, 95% CI = 18.4°C–20.9°C; 2023 mean = 18.4°C, 95% CI = 16.9°C–19.8°C). These temperatures closely mirrored those observed for corresponding fall arrival and ingress periods. Visual examination of the spring temperature profiles indicated departure in both years followed a sharp and persistent increase in temperatures over several consecutive days (Figure 8).

## 4 Discussion

Across taxa, many species reduce the fitness consequences of mistimed seasonal transitions by incorporating staging periods into their annual cycles. Such transitional phases allow individuals to delay otherwise irreversible seasonal shifts while continuing to monitor environmental cues, thereby better aligning movements with favorable conditions. (Chapman et al. 2015; Cohen et al. 2021; Fryxell and Sinclair 1988; Lacki et al. 2015; Schofield et al. 2018; Smith 2012; Tenger‐Trolander et al. 2023; Warnock 2010). Our results demonstrate that staging periods likely play a similar role in refugia‐dependent and migratory snakes, and other reptiles with similar life histories and spatial ecologies, where narrow thermal tolerances and short active seasons amplify the costs of phenological error. In our study system in west‐central Illinois, *C. horridus* remained within overwintering refugia for over 6 months, with fall and spring staging extending association with overwintering habitat to nearly 8 months and leaving only a brief window (~4 months) for active‐season activities. Compression of the active season would be expected to impose strong fitness constraints by limiting energy acquisition, growth, and reproduction (Aldridge and Brown 1995; Brown 2016; Martin 2002). Nevertheless, the consistent presence of prolonged staging periods indicates that these transitional phases are important, functioning as adaptive temporal buffers that decouple arrival from entry into overwintering refugia and emergence from departure to summer habitats. Collectively, staging appears to align migration and refugia use with fluctuating environmental conditions, highlighting an underexplored mechanism by which temperate reptiles mitigate phenological risk.

### 4.1 Spring Staging Period

*S*pring staging highlights how decoupling emergence from dispersal allows snakes to navigate thermally stochastic conditions during seasonal transitions. Northern populations of *C. horridus* and similar snakes face especially risky decisions in spring, first determining when to abandon the relative thermal and physical safety of refugia and subsequently when to initiate migration to summer habitats. Temperature has been identified as a key trigger for spring egress in *C. horridus* and other species (Andrews and Waldron 2017; Brown 1992; Jesper et al. 2023; Martin 1992), with general agreement that egress occurs near ~15°C and that activity outside refugia is minimal below ~10°C. Our results support such thresholds and further document a “stop–start” emergence pattern driven by brief warm periods interrupted by cold fronts, resulting in staggered emergence. Similar patterns have been reported in other studies of *C. horridus* , where peaks in emergence occurred whenever temperatures exceeded ~15°C (Brown 1992; Martin 1992). Although such stop–start emergence may appear to be a straightforward physiological response to fluctuating temperatures, prolonged asynchronous emergence has been noted in temperate reptiles without clear mechanistic explanation (Blouin‐Demers et al. 2000; DeGregorio et al. 2017; Gregory 1982). Our findings suggest this pattern reflects climatic stochasticity interacting with sequential decision‐making rather than noise or individual variation alone.

Once emerged, snakes must then decide when to depart from overwintering habitat and migrate to summer ranges. Dispersing too early risks exposure to cold conditions, whereas delaying departure reduces time available for foraging, growth, and reproduction. A novel result of our study indicates that snakes might balance these opposing pressures by responding to a second thermal threshold that, in our study of *C. horridus* , was approximately 5°C warmer than the egress threshold and which appears to have signaled the onset of consistently warm conditions suitable for summer activity. Few studies have explicitly examined the phenology or thermal cues of spring departure in snakes, but available evidence generally supports our findings, noting that migrations tend to begin with the onset of sustained, “summer‐like” temperatures (Martin 1992).

Although mean dates of spring dispersal differed slightly between years (3 days), departure occurred at similar temperatures, suggesting temperature is an important proximate cue, rather than solely a fixed photoperiodic or physiological trigger (Gregory 1982). Although thermal thresholds were consistent across both study years, additional multiyear data is required to fully evaluate the relative importance of thermal cues compared to other environmental or endogenous drivers. If temperature is indeed the cue, which our results suggest, staging periods may be truncated or absent altogether under consistently warm spring conditions, with snakes emerging and departing in rapid succession, as reported for southern populations or anomalously warm years (Martin 1992; Sealy 2002). Prolonged spring staging therefore appears to be a behavioral response to the stochastic thermal regimes characteristic of temperate springs and thus may be most pronounced at higher latitudes and elevations.

One unresolved question is why snakes emerge from refugia before conditions are suitable for departure and why they do not return to refugia during colder intervals, as suggested by some studies (e.g., Brown 1993). At a minimum, early emergence implies that adjacent south‐facing bluff habitats (e.g., crevices, talus, leaf litter) provide suitable thermal conditions during post‐egress cold periods. Alternative explanations include opportunistic feeding (Sealy 2002), shedding (Brown 1993), or physiological acclimation (Angilletta Jr. 2009). Although our data do not allow us to directly evaluate the relative importance of these mechanisms, we do not believe they represent dominant drivers of the prolonged staging behavior observed here. Prey availability and digestive temperatures were likely insufficient to support feeding, and anecdotal field observations suggested that most individuals delayed shedding until reaching summer habitats, consistent with previous findings (Carnes‐Mason and Beaupre 2023), although precise shedding timing was difficult to assess because snakes were frequently concealed. While basking during spring staging may facilitate physiological transitions, whether such acclimation is required prior to sustained activity remains unresolved. Resolving this question will require direct measurements of body temperature, metabolic state, and physiological performance during the overwintering and staging periods (Angilletta Jr. 2009).

### 4.2 Fall Staging Period

In contrast to spring, fall staging reflects an anticipatory response to approaching seasonal cooling. Snakes must ensure timely access to refugia before external temperatures fall below their thermal tolerances while delaying entry long enough to capitalize on remaining active‐season opportunities. Our results indicate that *C. horridus* consistently arrived at overwintering habitat at substantially warmer temperatures than those at which they ultimately ingressed, suggesting arrival and ingress are likely associated with distinct thermal cues. Notably, temperatures associated with fall arrival and ingress closely mirror those observed during spring departure and egress, respectively, indicating that both inbound and outbound movements are likely driven by similar thermal constraints across seasons rather than a single fixed cue. Taken together, the symmetry between spring and fall staging supports a full‐annual‐cycle perspective in which snakes use similar thermal information to regulate both outbound and inbound movements.

Snakes generally began arriving near refugia following temperature declines below ~15°C, corresponding to the voluntary thermal minimum reported for the species and similar to spring egress thresholds (Brown 1992; Jesper et al. 2023). However, because telemetry began once snakes approached overwintering bluff habitats near refugia (as identified in Jesper et al. 2024), we cannot determine the precise cues initiating fall migration from summer habitats. While temperature declines appear to accelerate final approach and ingress, another trigger likely initiates inbound movement earlier in the season. Regardless of arrival cues, most snakes did not immediately enter refugia upon arrival. Instead, they remained surface‐active near refugia until conditions necessitated ingress, resulting in prolonged fall staging during warm years (e.g., Fall 2021) and shorter staging during rapidly cooling years (e.g., Fall 2022). As in spring, the reasons for this delay remain uncertain. Individuals may attempt to extend active‐season activities, such as foraging, although we observed no feeding and would expect aphagia prior to ingress due to impaired digestion at low temperatures during brumation. Alternatively, fall staging may allow individuals to address physiological stressors, including infection or disease. Notably, the few individuals exhibiting abnormal winter activity appeared to emerge specifically to bask during warm periods, likely to facilitate immune function (Jesper et al. 2024; Nordberg and Cobb 2016).

## 5 Conclusions

For migratory, refugia‐dependent ectotherms in seasonal environments, successful migration requires precise timing without excessively compressing already short active seasons. (Harvey and Larsen 2020). Our results indicate that fall and spring staging periods function as adaptive, phenologically flexible temporal buffers that ensure reliable access to refugia and facilitate safe transitions across thermally stochastic periods. The timing and duration of staging periods appear flexible and closely linked to seasonal temperature fluctuations, supporting the idea that prolonged staging may be primarily a temperate‐latitude phenomenon.

The adaptive value of staging periods may become increasingly important under ongoing climate change. Rising mean temperatures accompanied by greater thermal variability (Lewis and King 2017) are expected to increase the frequency of misleading cues, such as transient warm periods followed by abrupt cold events (Wu et al. 2025). Under such conditions, strategies that decouple emergence from dispersal or arrival from ingress may buffer individuals against climate‐driven phenological traps. Conversely, disruption or loss of staging habitats could disproportionately increase mortality by forcing animals into premature or delayed transitions. Because staging habitats are adjacent to overwintering refugia, their degradation may disproportionately increase mortality by eliminating the behavioral flexibility required to respond to increasing climatic variability. Recognizing staging as a distinct and functionally important phase of the annual cycle therefore has important implications for predicting species responses to climate variability and for identifying critical habitats for conservation.

## Author Contributions

**Andrew C. Jesper:** conceptualization (equal), data curation (lead), formal analysis (lead), funding acquisition (supporting), investigation (equal), methodology (equal), project administration (equal), resources (supporting), software (supporting), supervision (lead), validation (lead), visualization (lead), writing – original draft (lead), writing – review and editing (lead). **Michael J. Dreslik:** conceptualization (equal), data curation (supporting), formal analysis (supporting), funding acquisition (supporting), investigation (equal), methodology (equal), project administration (equal), resources (supporting), software (supporting), supervision (supporting), validation (supporting), visualization (supporting), writing – original draft (supporting), writing – review and editing (supporting). **Scott A. Eckert:** conceptualization (equal), data curation (supporting), formal analysis (supporting), funding acquisition (lead), investigation (equal), methodology (equal), project administration (equal), resources (lead), software (supporting), supervision (supporting), validation (supporting), visualization (supporting), writing – original draft (supporting), writing – review and editing (supporting).

## Funding

This research was supported through private funding sources.

## Conflicts of Interest

The authors declare no conflicts of interest.

## Data Availability

We have archived the data supporting the findings of this study on Figshare (https://doi.org/10.6084/m9.figshare.31009993). To protect sensitive den and staging locations for a species of conservation concern, we do not publicly share raw GPS spatial coordinates. Instead, we provide all processed data used in the analyses. See the Figshare description for further information.
